# Arabic Version of the Electronic Health Literacy Scale in Arabic-Speaking Individuals in Sweden: Prospective Psychometric Evaluation Study

**DOI:** 10.2196/24466

**Published:** 2021-03-22

**Authors:** Josefin Wångdahl, Karuna Dahlberg, Maria Jaensson, Ulrica Nilsson

**Affiliations:** 1 Department of Public Health and Caring Sciences Uppsala University Uppsala Sweden; 2 Department of Neurobiology, Care Sciences and Society Karolinska Institute Stockholm Sweden; 3 School of Health Sciences Faculty of Medicine and Health Örebro University Örebro Sweden; 4 Department of Perioperative Medicine and Intensive Care Karolinska University Hospital Stockholm Sweden

**Keywords:** eHealth, digital health literacy, eHEALS, health literacy, internet, psychometrics, evaluation, migrants, refugees, Arabic

## Abstract

**Background:**

Health information is often communicated through the internet. It is vital for the end user to have a range of digital skills as well as understand the information to promote their health. There is a valid and reliable 8-item instrument, the Electronic Health Literacy Scale (eHEALS), that evaluates these skills. The number of Arabic-speaking people migrating to Sweden and to other parts of the world is increasing due to unstable military and political situations in their countries of origin. Poor health and limited health literacy have been described in this population in Sweden. Still, to our knowledge, an Arabic version of eHEALS has not been tested for validity or reliability. Thus, Arabic-speaking populations in Sweden cannot be included in studies measuring eHealth literacy, which does not support equal treatment in health care.

**Objective:**

The aim of this study was to translate and adapt the original English eHEALS version into Arabic and to evaluate its psychometric properties.

**Methods:**

The eHEALS was rigorously translated, adapted, and evaluated for content validity. We conducted prospective psychometric evaluation with natively Arabic-speaking participants living in Sweden. Construct validity, factor structure, internal consistency, and test-retest reliability were evaluated using Spearman correlation, principal component analysis, Cronbach α, and weighted quadratic Cohen κ, respectively.

**Results:**

The study population consisted of Arabic-speaking participants (n=298; age: mean 41.8 years, SD 10.5). Construct validity was supported with weak and moderate correlations. Principal component factor analysis revealed a one-factor structure. Internal consistency was high (Cronbach α=0.92); test-retest reliability was acceptable (weighted quadratic Cohen κ=0.76). Evaluation indicated that eHealth literacy threshold values should be dichotomized (limited and sufficient) rather than trichotomized (inadequate, problematic, and sufficient).

**Conclusions:**

The Arabic version of eHEALS, a unidimensional scale that is valid and reliable for measuring eHealth literacy among natively Arabic-speaking people in Sweden, was found to be acceptable and feasible in a general population.

## Introduction

Arabic is 1 of 6 official languages of the United Nations and the official language of more than 20 countries. Unstable political and military situations have led to forced displacement for many people in some of these countries. In 2019, Arabic was one of the most widely spoken languages among refugees [1] (ie, someone who has been forced to flee their country because of persecution, war or violence; has a well-founded fear of persecution for reasons of race, religion, nationality, political opinion or membership in a particular social group; and that most likely cannot return home or are afraid to do so [2]) worldwide. Most refugees (approximately 6.6 million) came from Syria, which is also the most common country of origin for refugees overall. In Sweden, approximately 200,000 refugees, of which most were Syrian, speak Arabic [3]. A large number of refugees had less than good self-assessed health [4,5] and impaired psychological well-being [4-7]. Smoking, physical inactivity, and obesity (or being overweight) were also quite common [5]. At the same time, up to 73% of Arabic speaking refugees in Sweden refrain from seeking necessary health care, due to language problems, having the idea that help will not be given, or a lack of knowledge about where to go [4,5,7]. Arabic-speaking refugees from Iraq and Syria are overrepresented in the COVID-19 infections in Sweden [8], partly due to a lack of information [9].

One social determinant that may partly contribute to people’s health is health literacy [10], which “is linked to literacy and entails people’s knowledge, motivation and competences to access, understand, appraise and apply health information in order to make judgements and take decisions in everyday life concerning health care, disease prevention and health promotion to maintain or improve quality of life during the life course [11].” Studies show that approximately 60% of all Arabic-speaking refugees in Sweden have limited health literacy [4,5,12], a proportion that has been found among Syrian refugees in Turkey as well [13]. Among Arabic-speaking refugees in Sweden, associations have been found between limited health literacy and poor self-assessed health and between impaired psychological well-being and having refrained from seeking health care [14]. Furthermore, associations have been found between limited health literacy and poor communication, as well as with perceptions of receiving little new knowledge and help from the health examination for asylum seekers [4].

A specific form of health literacy is eHealth literacy which “is the ability to seek, find, understand, and appraise health information from electronic sources and apply the knowledge gained to addressing or solving a health problem [15].” The term eHealth (ie, electronic health) came into use in the year 2000, but there is no clear definition of it [16]. The internet is an important resource for health-related information and health services. To navigate and find this information requires a range of digital skills [17], which is challenging for both patients and health care staff [18]. Another potential challenge is limited language proficiency, which can result in lower understanding of health information and instructions [14,19,20]. Furthermore, there are different types of online health information of varying quality that people need to compare and evaluate. There are also rapid changes in both care routines and technology, and health information is updated frequently—yesterday’s health information may not be good practice today [15] or may not even be in practice at all.

To be able to draw conclusions about limited eHealth literacy among people who have migrated from countries where another language is spoken, and consequently, may have limited competencies in the official language of the country in which they live, a reliable and valid questionnaire is needed in a language in which they are fluent. The 8-item eHealth Literacy Scale (eHEALS) measures a broad range of eHealth literacy skills [15,21] on a 5-point Likert scale (from strongly agree to strongly disagree) eHealth literacy is classified using the sum score as inadequate, 8-20; problematic, 21-26; or sufficient, 27-40 [17]. The eHEALS is available in a range of languages [15,17,22-28] but not yet in Arabic. Psychometric testing of eHEALS indicates that it is a reliable and valid instrument [15,17,27,29-31] but also that its validity requires further investigation [28] and that the newly adapted thresholds need to be confirmed [17]. Consequently, the aim of this study was to translate and adapt the eHEALS into an Arabic version and to evaluate its psychometric properties.

## Methods

### Study Design and Participants

This was a prospective psychometric evaluation study that included 3 phases: translation, content validity testing, and psychometric evaluation. Data collection for phases 1 and 2 took place in April 2019, and data collection for phase 3 took place from May to September 2019 [32]. The project was approved by the Regional Ethical Review Board in Stockholm, Sweden (No. 2019/5:1) and was conducted in accordance with the 1964 Declaration of Helsinki and its subsequent amendments. All participants were informed in verbal and written formats about the purpose of the study, its procedures, and that participation was voluntary and withdrawal was possible at any time. The participants were given the guarantee that their information would be kept confidential and stored securely.

### Phases

#### Translation

The translation process was guided by the COSMIN (Consensus-Based Standards for the Selection of Health Measurement Instruments) study design checklist for Patient-reported outcome measurement instruments [33]. Permission was obtained from the creator of the eHEALS [15]. One translator, multilingual in Arabic, English, and Swedish and with Arabic as their native language, translated the original English version of eHEALS into Arabic. The translator was not a professional translator but had a high reputation in translating health surveys. Instructions to the translator were that plain language should be used and that a young person should easily be able to understand the translation; in other words, that the items should be short, easy to understand, and not contain difficult words [34]. The focus was on maintaining the meaning of the items but also to make them easy to understand and answer for people of varying educational and health literacy levels.

The translated eHEALS (Arabic) version was compared with the Swedish version (Sw-eHEALS [17]) to ensure that the Arabic version was in line with both original English and Swedish versions. The translator and one researcher went through each item together to verify its content, the use of plain language, and similarity. Some simplifications and adjustments of the language were made.

Four individuals who were fluent in Arabic, English, and Swedish were recruited to form a committee to examine the quality of the translation [35]—2 members had previous experience in translating health survey questions to Arabic, one of whom was also an experienced educator working with individuals who have migrated to Sweden, and 2 members had experience in health communication as nurses, one of whom also worked as a research assistant in this study. The committee members worked independently and were given the Arabic translation, the original English, and the Swedish versions of eHEALS and asked to comment on spelling, grammar, whether plain language was used, and to what extent the Arabic version was consistent with the other 2 eHEALS versions. Feedback and suggestions for improvement were received by email. One of the researchers and the research assistant discussed the feedback, which resulted in some minor changes in the wording of items and response options. The English-version term *health resources* was translated as *health information* in Arabic, partly because the meaning of the word better matches and partly because it is more common in everyday Arabic. *Health resources* has also been translated as *health information* in the Swedish version of eHEALS [15,17].

The Arabic version was then tested by 6 Swedish- and Arabic-speaking laypeople (Table 1) recruited purposively and through snowball sampling [36] by one of the researchers and the research assistant. Written and verbal information about the study was provided, and a mix of gender, age, and educational levels was sought. Based on the feedback from the participants, one of the researchers and the research assistant discussed changes that might improve the Arabic version; thereafter, the research assistant made some modifications, which consisted mainly of grammar corrections and determining which word should be in bold. Finally, a professional translator was given the Arabic version with the English and Swedish versions, in order to compare the 3 versions. The translator’s verdict was that the Arabic version matched the other 2 versions in terms of purpose and content.

**Table 1:** Demographics of the test group (n=6).

Characteristics		Value
**Gender, n**		
	Male	3
	Female	3
Age (in years), mean (range)		38 (24-52)
**Country of birth, n**		
	Syria	2
	Iraq	1
	Algeria	1
	Palestine	1
	France	1
Number of years lived in Sweden, mean (range)		16 (1-30)
**Educational levels, n**		
	10-12 years	1
	Graduated from university	5

#### Content Validity

Ar-eHEALS (Multimedia Appendix 1) content validity, the degree to which the content of an instrument is an adequate reflection of the construct that it is meant to measure [34,37], was evaluated through individual interviews with the test group composed of the 6 Swedish- and Arabic-speaking laypeople who participated in phase 1. The participants were told to think aloud during as they completed the Ar-eHEALS questionnaire and indicate whether anything felt problematic. They were also asked to argue how they were reasoning when answering the each of the 8 items.

The meaning of health information was interpreted differently; some participants mentioned public health information (available on, for example, Sweden’s 24-hour helpline 1177 and Family Life, a parent forum on the internet). Other participants mentioned health information in scientific articles and reports addressed to health care professionals. In some cases, health information from friends and health care was mentioned (ie, information that was not available on internet).

The importance of, and difficulty with, health information source criticism was raised in connection with 4 out of 8 items (items 1, 3, 7, 8); participants concluded that talking to and getting information directly from a doctor was best. Whether or not a user had education in health or health care was brought up by some participants, in connection with 2 out of 8 items (items 6 and 8), as a factor regarding to what extent the user agrees with the statement (ie, assessing and using health information appropriately). Participants’ reasoning about each item corresponded with the response alternative they chose and what the item aimed to measure. Five out of 8 items (items 3-7) were perceived to be closely related to the item immediately preceding the item to which they answered, which in turn made it difficult to distinguish between them. The participants mostly chose the same response alternative for the items that they thought were similar. However, all items were experienced as short and easy to understand, despite the similarities mentioned above. The response alternatives were perceived as good and no changes were made to the questionnaire after the content validity evaluation.

#### Psychometric Evaluation

##### Participants, Settings, and Data Collection

**Figure 1:**
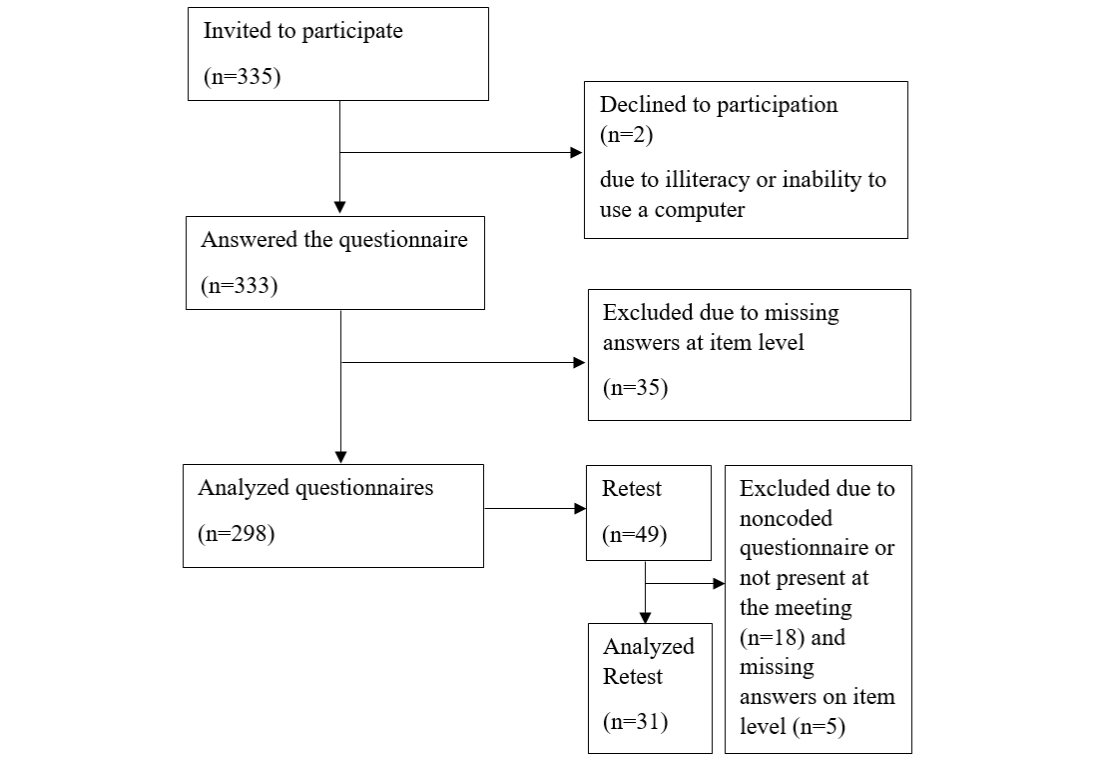
Data collection flowchart.

A sample size of 300 was considered to be appropriate [33,38]. The inclusion criteria for participation were age 18 years or older, a native speaker of Arabic, and available on the day of the data collection. The first author visited 9 different arenas: courses in civic orientation for newly arrived refugees in Sweden, fast tracks from newly arrived academics at universities, an Arabic language school, a theatre, a parent support groups, and 2 informal Arabic-language networks in order to recruit a range (gender, age, and educational levels) of participants Of the 335 people invited to take part in the study, 2 declined to participate due to illiteracy or lack of computer skills, and 35 were excluded from the study because they lacked a valid eHEALS sum score, which resulted in a study population of 298 participants (Figure 1).

To analyze test-retest reliability of Ar-eHEALS and HLS-EU-Q16, 49 participants were invited to answer the questionnaire twice with a 1-week interval. As the sample size needed for test-retest is much smaller than for testing many other forms of validity, a sample size of 25 people for the retest was considered appropriate [39]. However, in order to recruit a range and account for attrition, 49 people were asked to participate in the test-retest. To minimize dropout, participants in the test-retest groups were recruited in 2 of the groups having regular weekly meetings. To be able to compare answers from the test and the retest on individual level, the participants marked their questionnaires with a code consisting of the first 3 letters of their mothers’ names and the year she was born. There was thus no need to print personal numbers and the participants remained anonymous. Of the 49 people who were asked to participate in the test-retest group, 18 people were not present at the second measurement or did not fill in the personal code to enable paring with the first questionnaire, and 5 people had at least 1 invalid Ar-eHEALS sum score; therefore, a total of 31 participants were included in the test-retest analysis (Figure 1).

##### Study Questionnaires and Additional Questions

Participants were given the Ar-eHEALS questionnaire, the short version of the Arabic version of the Health Literacy Survey European Questionnaire (HLS-EU-Q16) [40], questions about demographic information (age, gender, education levels, country of birth, and years lived in Sweden) and questions about health and use of the internet. The HLS-EU-Q16 consists of 16 items measuring comprehensive health literacy—perceived personal skills in finding, understanding, judging, and applying health information in order to maintain and improve health [40]. The items were answered on a 4-point Likert scale ranging from very difficult to very easy. A sum score (ranging from 0 to 16) was calculated, and self-perceived comprehensive health literacy was classified into 3 levels: 0-8, inadequate, 9-12, problematic, and 13-16, sufficient [40]. The general self-perceived health question “How do you assess your overall health status?” had “very poor, poor, fair, good, or very good” as response options [14,32,41,42]; the question “How useful is the Internet in helping you make decisions about your health?” had “not useful at all, not useful, unsure, useful, or very useful” as response options and was used to measure the usability of the internet. Importance of the internet was measured by the question “How important is it for you to be able to access health resources on the Internet?” with “not important at all, not important, unsure, important, and very important” as response options [15,17]. The question “How often do you use the Internet?” had “every day (or almost every day), several days a week, around once a week, less than day a week and never (or almost never)” as response options [17,28].

##### Psychometric Testing

The psychometric testing was guided by COSMIN guidelines [33,34,37,43]. Construct validity is the degree to which results from an instrument are consistent with a hypothesis [33]. In previous studies on health literacy, positive associations have been found between limited health literacy and high age [17,44-47], poor health [14,17,45,48-50], and low levels of education [17,47,51,52]. The hypotheses used in this study to evaluate the construct validity were that there is a negative correlation between Ar-eHEALS sum score and age, and there is a positive correlation between Ar-eHEALS level and education as well as between Ar-eHEALS and self-perceived general health. Other hypotheses were based on our earlier psychometric evaluation of the Swedish version of eHEALS [17], showing positive correlations between Ar-eHEALS versus interest in and level of internet use [17], the HLS-EU-Q16 sum score, and items A, K, L, and M in HLS-EU-Q16 focusing on health literacy and the Internet.

To confirm the factor structure of Ar-eHEALS principal component factoring analysis was used [53]. Cronbach α was used to assess the average correlation for the sum score of Ar-eHEALS [33].

Test-retest reliability (longitudinal reliability) was examined by calculating weighted quadratic Cohen κ coefficients [33,37].

To examine floor and ceiling effects (ie, the number of participants who choose the lowest or highest possible scores when answering), the proportion of participants who had chosen different answer alternatives was calculated. If more than 15% of a study population had chosen the lowest or highest possible score, floor or ceiling effects could be considered to be a problem [43].

The Ar-eHEALS scores were categorized according to range as inadequate (8-20), problematic (21-26), and sufficient (27-40). A dichotomization was also performed according to range: limited (8-26) and sufficient (27-40) [17].

##### Statistical Analysis

Data are presented as mean, standard deviation, number, percentage, or range. Spearman rank was used to analyze the correlation between the mean sum Ar-eHEALS score and the following: HLS-EU-Q16, self-perceived health, level of education, and age. A correlation coefficient magnitude between 0 and 0.1 was considered to be negligible, between 0.1 and 0.39 was considered to be weak, between 0.4 and 0.69 was considered to be moderate, between 0.7 and 0.89 was considered to be strong, and between 0.9 and 1.0 was considered to be very strong [54]. Cronbach α with a range of 0.70 to 0.95 was considered acceptable [34,55]. The weighted quadratic Cohen κ coefficient, with an accepted value of ≥0.70, was used to measure test-retest reliability [34,56]. To test thresholds, The Friedman test was used to analyze differences between Ar-eHEALS and HLS-EU-Q16 in terms of numbers of patients with inadequate, problematic, and sufficient health literacy; the Wilcoxon signed-rank test was used to analyze the threshold for limited (ie, inadequate and problematic) and sufficient health literacy. The chi-square test was used to analyze differences in gender, and the student *t* test was used to analyze differences in age. The Wilcoxon signed-rank test was used to analyze differences in educational levels, general self-perceived health, and Ar-eHEALS levels between participants with the same levels of health literacy on both the Ar-eHEALS and HLS-EU-Q16 compared with those with different levels. All data were analyzed using SPSS statistical software (version 24.0 for Windows; IBM Corp). Two-tailed *P* values <.05 were considered significant.

## Results

### Participants

The mean age was 41.8 years (SD 12.5), there was a higher proportion of males (180/292, 61.6%), 75% (222/296) had at least 10 years of education, and 66.4% (198/298) perceived their own general health as good or better (Table 2). On average, the participants had lived in Sweden for 9 years (range 0-38, SD 8.2). Less than half (111/289, 38.4%) had sufficient health literacy, and the mean sum score of Ar-eHEALS was 28.1 (SD 6.1). Most participants reported that they use the internet almost every day (255/297, 85.9%), that they think the internet is useful or very useful (205/298, 68.8%), and that the internet is important or very important (205/296, 69.2%). No statistically significant differences regarding gender (*P*=.19), age (*P=*.22), or education level (*P*=.93) could be found between participants who were included and those who were excluded due to missing Ar-eHEALS sum scores. Nor was any pattern of structural problems found, in terms of difficulty in responding to certain items.

**Table 2:** Demographics of the respondents with a valid eHEALS sum score and the test-retest group.

Characteristics		All (n=298)^a^	Test-retest group (n=31)
**Gender, n (%)**			
	Male	112 (38.4)	9 (29.0)
	Female	180 (61.6)	22 (71.0)
**Age in years**			
	Mean (SD)	41.8 (12.5)	47.8 (10.0)
	Range	21-77	30-68
**Country of birth**			
	Syria	179 (60.1)	13 (41.9)
	Iraq	65 (21.8)	11 (35.5)
	Sudan	14 (4.7)	0 (0)
	Other country	40 (13.4)	5 (16.1)
**Number of years lived in Sweden**			
	Mean (SD)	9.4 (8.2)	11.2 (10.9)
	Range	0-38	1-30
**Highest education level, n (%)**			
	None	5 (1.7)	1 (3.2)
	1-6 years	24 (8.1)	2 (6.5)
	7-9 years	45 (15.2)	5 (16.1)
	10-12 years	65 (22.0)	6 (19.4)
	Graduated from university	157 (53.0)	17 (54.8)
**General self-perceived health, n (%)**			
	Very poor	6 (2.0)	1 (3.2)
	Poor	19 (6.4)	0 (0.0)
	Fair	74 (24.9)	13 (41.9)
	Good	127 (42.8)	12 (38.7)
	Very good	71 (24.9)	5 (16.1)
**HLS-EU-Q16^b^, n (%)**			
	Inadequate	64 (22.1)	4 (13)
	Problematic	114 (39.4)	10 (32)
	Sufficient	111 (38.4)	17 (55)
**Ar-eHEALS^c^**			
	Mean (SD)	28.1 (6.1)	27.8 (7.1)
	Range	8-40	10-40
**Frequency of internet use, n (%)**			
	Never	5 (1.7)	1 (3.2)
	Less than 1 day 1 week	5 (1.7)	0 (0.0)
	Approximately 1 day a week	7 (2.4)	0 (0.0)
	Several days a week	25 (8.4)	3 (9.7)
	Every day	255 (85.9)	27 (87.1)
**Usability of the internet, n (%)**			
	Not useful at all	8 (2.7)	1 (3.2)
	Not useful	21 (7.1)	3 (9.7)
	Unsure	62 (20.9)	3 (9.7)
	Useful	126 (42.6)	16 (51.6)
	Very useful	79 (26.7)	8 (25.8)
**Importance of the internet, n (%)**			
	Not important at all	11 (3.7)	2 (6.5)
	Not important	18 (6.1)	2 (6.5)
	Unsure	62 (20.9)	6 (19.4)
	Important	120 (40.5)	13 (41.9)
	Very important	85 (28.7)	8 (25.8)

^a^Missing responses (gender, n=6; age, n= 11, number of years lived in Sweden, n=77; highest education level, n=2; HLS-EU-Q16, n=9; frequency of internet use, n=1; importance of the internet, n=2) were not included in the denominator when calculating percentages.

^b^HLS-EU-Q16: Health Literacy Survey European Questionnaire.

^c^Ar-eHEALS: eHealth Literacy Scale.

### Construct Validity

No correlation was found between the Ar-eHEALS sum score and age. A weak positive correlation was found between Ar-eHEALS and the following: education level, self-perceived health, frequency of internet use, and item A in HLS-EU-Q16. A moderate positive correlation was found between Ar-eHEALS and the following: usability of the internet; importance of the internet, HLS-EU-Q16 sum score; and items K, L, and M in HLS-EU-Q16 (Table 3).

**Table 3:** Spearman correlations between the Ar-eHEALS sum score and demographic characteristics, questions, and questionnaires.

Variable	Spearman ρ	*P* value
Age	–0.10	.19
Education level	0.25	<.001
Self-perceived health	0.30	<.001
Usability of the internet	0.43	<.001
Importance of the internet	0.42	<.001
Frequency of internet use	0.14	.01
HLS-EU-Q16^a^ sum score	0.45	<.001
HLS-EU-Q16 item A: Find information on treatments of illnesses that concern you	0.31	<.0001
HLS-EU-Q16 item K: Judge if the information on health risks in the media is reliable	0.41	<.001
HLS-EU-Q16 item L: Decide how you can protect yourself from illness based on information in the media	0.44	<.001
HLS-EU-Q16 item M: To understand informa­tion in the media on how to get healthier	0.46	<.001

^a^HLS-EU-Q16: Health Literacy Survey European Questionnaire, 16-item.

### Reliability

#### Internal Consistency

Factor analysis showed that the Kayser-Meyer-Olkin measure of sampling adequacy for the analysis was good (0.88, *P*<.001). Principal component analysis resulted in a one-factor solution with an initial eigenvalue of 5.0, accounting for 62.7% of the variance. The scree plot also showed a one-factor structure. All items had accepted loadings ranging from 0.63 to 0.86 (Table 4). Cronbach α=0.92 was considered acceptable as it was within the acceptable range of 0.70 to 0.95.

**Table 4:** Principal component analysis and weighted quadratic Cohen κ for the Arabic version of the eHealth Literacy Scale sum score or individual items.

Variable	Factor loadings	Weighted quadratic Cohen κ
eHEALS^a^ sum score	N/A^b^	0.76
Item 1: I know what health resources are available on the Internet	0.63	0.78
Item 2: I know where to find helpful health resources on the Internet	0.83	0.73
Item 3: I know how to find helpful health resources on the Internet	0.86	0.83
Item 4: I know how to find helpful information^c^ on the Internet	0.83	0.62
Item 5: I know how to use the health information^c^ I find on the Internet to help me	0.83	0.68
Item 6: I have the skills I need to evaluate the health resources I find on the Internet	0.78	0.47
Item 7: I can tell high quality health resources from low quality health resources on the Internet	0.79	0.64
Item 8: I feel confident in using information from the Internet to make health decisions	0.77	0.47

^a^Ar-eHEALS: Arabic version of the eHealth Literacy Scale.

^b^N/A: not applicable.

^c^The term *health resources* was used instead of *health information*, which is used in the original version [15].

#### Test-Retest Reliability

A total of 31 participants with a mean age of 47.8 years (SD 10.0) were included in the test-retest; 29.0% (9/31) were male, 74.2% (23/31) had at least 10 years of education, and 54.8% (17/31) perceived their own general health as good or better (Table 2). The majority (17/31, 54.8%) had sufficient comprehensive health literacy, and the mean sum score of Ar-eHEALS was 27.8 (SD 7.1). Most participants reported that they use the internet almost every day (27/31, 87.1%), that they think the internet is useful or very useful (24/31, 77.4%), and that the internet is important or very important (21/31, 67.7%). Test-retest reliability of the Ar-eHEALS sum score was acceptable (Cohen κ=0.76, *P*<.001). At the items level, test-retest reliability was acceptable for 4 of 8 items (Cohen κ=0.73-0.83; Table 4).

#### Floor and Ceiling Effects

No floor and ceiling effects were found for the Ar-eHEALS; 3.7% (11/298) had the highest possible sum score, and 1.0% (3/298) had the lowest possible sum score.

#### Thresholds

When comparing numbers of participants with inadequate, problematic, and sufficient health literacy (HLS-EU-Q16) with eHealth literacy (Ar-eHEALS), there were statistically significant differences depending on which scale was used (*P*<.001). Higher proportions of inadequate (64/289, 21.5%) and problematic (114/289, 39.4%) were found for health literacy than were found for eHealth literacy; inadequate (26/289, 8.9%) and problematic (83/289, 28.7%). The opposite was found for sufficient health literacy (111/289, 38.4%) compared to sufficient eHealth literacy (180/289, 62.2%). When dichotomizing Ar-eHEALS and HLS-EU-Q16 into limited (inadequate and problematic combined) and sufficient health literacy, there was a significantly greater proportion of participants who scored the same level of health literacy on both questionnaires compared to participants who had different levels (same: 184/289, 63.7%; different: 105/289, 36.3%; *P*<.001; Table 5). There were no significant differences in age (*P*=.52*)*, gender (*P=*.20*)*, educational level (*P*=.77*)*, or general self-perceived health (*P=*.11) between these 2 groups.

**Table 5:** Distribution of participants scoring sufficient resp. limited health literacy and e-health literacy and participants scoring different levels depending on which questionnaire.

Health literacy (HLS-EU-Q16^a^)	eHealth literacy (Ar-eHEALS^b^), n (%)^c^	
	Limited	Sufficient
Limited	91 (31.5)	87 (30.1)
Sufficient	18 (6.2)	93 (32.2)

^a^HLS-EU-Q16: Health Literacy Survey European Questionnaire.

^b^Ar-eHEALS: eHealth Literacy Scale.

^c^Percentage of all participants with responses (n=289).

## Discussion

### Principal Findings

The results of this psychometric evaluation support the use of Ar-eHEALS to measure the self-reported eHealth literacy of Arabic-speaking people living in Sweden. The majority of the participants (179/298, 60.1%) came from Syria, which reflects the general population of the newly arrived refugees in Sweden [57]. The translation process is of great importance, especially when measuring a phenomenon such as eHealth literacy, in order to take cultural adaptation into consideration. Kalfoss [58] pointed out that some of the difficulties encountered in the translation process may be due to the forward translations where the words are translated too closely (ie, word-for-word translation, meaning that the translation focused on the words and not the meaning of the question). This was an aspect in our translation process regarding the word *health resources*. Even though a lot of effort was invested in this translation, it was obvious that the word that replaced *health resources* (ie, *health information*) was interpreted differently by the participants during the evaluation of content validity. However, *health information* is a more common term in everyday Arabic than *health resources*, and the translators indicated that *health information* is a more appropriate concept in Arabic. The creator of the original [15] was also contacted; we were informed that there have been similar problems with the concept when translating it into other languages.

Translating, adapting, and validating a questionnaire for practice or research is a time-consuming process that requires careful planning and a rigorous methodological approach to produce a reliable and valid measure of the concept of interest in the target population [58]. In the translation process, it is necessary to ensure that the forward translator has expertise in the specific topic (ie, in this case the target population) and in the construct [33]. In this study, only one forward translator was used. However, the committee that examined the quality of the translation consisted of 4 people who were multilingual in Arabic, English, and Swedish, with experience in translating health surveys, data collection in Arabic, health communication, or in education for people who have migrated. Lastly, the translation process involved laypeople and an expert for feedback on wording. This step is particularly important when an understanding of the items is vital, for example, in a questionnaire measuring literacy skills such as that measuring eHealth literacy in our study.

In the psychometric evaluation, the Ar-eHEALS and the Sw-eHEALS [17] were found to have a one-factor structure (ie, unidimensionality). The eHEALS was originally proposed to have a one-factor structure [15], which has been supported by substantial evidence which has been supported by substantial evidence irrespective of which test theory—classical or modern—was used [22,24-26,28,59-63]. The unidimensionality indicates that all the items measure a single underlying construct, in this case eHealth literacy. One can argue that confirmatory factor analysis should be more appropriate; however, principal component factoring analysis loadings are sometimes closer approximations of the true factor loadings than the loadings produced by confirmatory factor analysis. Another difference between is that confirmatory factor analysis explains a correlation matrix, whereas principal component factoring analysis identifies the major sources of variation in data. [53]. However, the sample size for this study (n=298) was appropriate, since a sample size ≥100 participants is appropriate for conducting psychometric evaluations such as internal consistency analysis [33].

According to the context-specific nature of eHealth literacy skills [15], a moderate correlation was found between eHealth literacy measured by Ar-eHEALS sum score and health literacy measured by HLS-EU-Q16 sum score and 3 of the 4 HLS-EU-Q16 items focusing on health literacy and the internet. This is in line with the findings of previous studies [17,64] showing a relationship between eHealth literacy and health literacy. There was a moderate correlation between the Ar-eHEALS and perceptions of the usability (ρ=0.43) and between the Ar-eHEALS and perceptions of the importance of using the internet (ρ=0.42), and there was a weak correlation (ρ=0.30) between Ar-eHEALS and self-perceived health, in line with findings for Sw-eHEALS [17]. A weak correlation between the Norwegian version of eHEALS and health status has been reported [64]; however, the question about self-perceived health used in this study has not yet been validated. On the other hand, it has been argued that self-perceived health is credible indicator reflecting a person’s subjective general perception of health [41].

Ar-eHEALS demonstrated a high reliability (Cronbach α=0.92), which is in line with finding for other language versions of eHEALS (Cronbach α ≥0.88) [22,24,26,59-63] and moderate stability over time (weighted quadratic Cohen κ=0.76 coefficient for Ar-eHEALS), which was acceptable and higher than that of the Norwegian eHEALS (Cohen κ=0.61) [64] but lower than of the Swedish version of eHEALS (Cohen κ=0.86) [17].

Use of inadequate, problematic, and sufficient levels, as previously used for Sw-eHEALS [17], could not be confirmed for the Ar-eHEALS. However, when dichotomizing the threshold into sufficient and limited, the thresholds seemed to be relevant. One can argue that for a short questionnaire such as eHEALS with 8 items, 3 threshold levels is too many and may threaten the sensitivity and specificity of the questionnaire. The most important purpose must be to identify those individuals and groups who suffer from limited eHealth literacy. Nevertheless, the threshold levels for eHEALS require further evaluation in other populations and in other language versions [17].

The purpose of our study was to develop an Arabic version of eHEALS in order to include Arabic-speaking individuals living in Sweden in future eHealth literacy research. We do believe that more knowledge about associations between eHealth literacy and health outcomes, about to what extent disease prevention and health care efforts are beneficial for Arabic-speaking Swedish residents with different levels of eHealth literacy, may be important in efforts to reduce health inequalities. Since approximately 315 million people worldwide are Arabic speaking, we also think that Ar-eHEALS can be used globally, if further validated in the specific country and context in which it is to be used.

### Limitations

This study was not without limitations. One limitation is that 5 out of 6 participants in the content validity test group had university degrees. People with lower levels of education might understand and interpret the items differently. Another limitation is that it is not clear to what *health information* referred (whether it represented the Arabic or Swedish concept of health information). We suggest that in future use of the Arabic version, the concept of heath information should be specified. The sample included in this study may not be representative of all Arabic-speaking individuals in Sweden. However, the participants included were recruited from different arenas, and included different ages, genders, and levels of education. Furthermore, there were some item for which information was missing, such as for number of years lived in Sweden (77/298, 25.8% missing). One reason for this could be that those respondents were born in Sweden and therefore did not find it relevant to answer this item. Because this missing information was considered to not influence the psychometric evaluations and because all items in the Ar-eHEALS were answered, we decided to include these questionnaires in the analysis.

### Conclusion

The Ar-eHEALS has rigorously and successfully been translated and culturally adapted for an Arabic-speaking population in Sweden. The psychometric testing showed that the Ar-eHEALS is valid and reliable and can be used to assess eHealth literacy among Arabic-speaking people in Sweden. Furthermore, it indicates that sum scores should be dichotomized (into sufficient and limited eHealth literacy), but further evaluation is needed.

## Appendix

Multimedia Appendix 1 Arabic eHEALS version.

## Abbreviations

Ar-eHEALS: Arabic version of eHEALS
eHEALS: eHealth Literacy Scale
HLS-EU-Q16: Health Literacy Survey European Questionnaire–16
Sw-eHEALS: Swedish version of eHEALS

## References

[1] (2019). Global Trends: Forced Displacement in 2019. UNHCR The UN Refugee Agency.

[2] (2011). UNHCR Resettlement Handbook and Country Chapters. UNHCR The UN Refugee Agency.

[3] (2020). Beviljade uppehållstillstånd 2009-2019 [Granted residence permits 2009-2019]. The Swedish Migration Board.

[4] Wångdahl J, Lytsy P, Mårtensson L, Westerling R (2018). Poor health and refraining from seeking healthcare are associated with comprehensive health literacy among refugees: a Swedish cross-sectional study. Int J Public Health.

[5] Zdravkovic S, Cuadra Björnegren C (2016). Kartläggning av nyanländas hälsa [Mapping of the newcomers' health]. Malmö University.

[6] Tinghög P, Malm A, Arwidson C, Sigvardsdotter E, Lundin A, Saboonchi F (2017). Prevalence of mental ill health, traumas and postmigration stress among refugees from Syria resettled in Sweden after 2011: a population-based survey. BMJ Open.

[7] Lecerof SS, Stafström M, Emmelin M, Westerling R, Östergen P (2017). Findings from a prospective cohort study evaluating the effects of International Health Advisors' work on recently settled migrants' health. BMC Public Health.

[8] (2020). Veckorapport covid-19 vecka 15 publicerad 17 april 2020 [Weekly report covid-19 week 15 published 17 April]. The Public Health Agency in Sweden.

[9] Esaiasson P, Johansson B, Ghersetti M, Sohlberg J (2020). Kriskommunikation och segregation i en pandemi. Hur boende i utsatta områden informerade sig om coronaviruset våren 2020. Arbetsrapport nr 84 [Crisis communication and segregation in a pandemic. How residents in vulnerable areas found out about the coronavirus in the spring of 2020. Work report no. 84]. Department of Journalism, Media and Communication, Gothenburg University.

[10] Kickbusch IS (2001). Health literacy: addressing the health and education divide. Health Promot Int.

[11] Sørensen Kristine, van den Broucke Stephan, Fullam J, Doyle G, Pelikan J, Slonska Z, Brand H, (HLS-EU) Consortium Health Literacy Project European (2012). Health literacy and public health: a systematic review and integration of definitions and models. BMC Public Health.

[12] Wångdahl J, Lytsy P, Mårtensson L, Westerling R (2014). Health literacy among refugees in Sweden - a cross-sectional study. BMC Public Health.

[13] Assessing the health literacy and health communication needs of Syrian refugees in Turkey. World Health Organization Regional Office for Europe.

[14] Wångdahl J, Lytsy P, Mårtensson L, Westerling R (2015). Health literacy and refugees' experiences of the health examination for asylum seekers - a Swedish cross-sectional study. BMC Public Health.

[15] Norman CD, Skinner HA (2006). eHEALS: The eHealth Literacy Scale. J Med Internet Res.

[16] Eysenbach G (2001). What is e-health?. J Med Internet Res.

[17] Wångdahl J, Jaensson M, Dahlberg K, Nilsson U (2020). The Swedish version of the Electronic Health Literacy Scale: prospective psychometric evaluation study including thresholds levels. JMIR Mhealth Uhealth.

[18] Tan SS, Goonawardene N (2017). Internet health information seeking and the patient-physician relationship: a systematic review. J Med Internet Res.

[19] Kirmayer LJ, Narasiah L, Munoz M, Rashid M, Ryder AG, Guzder J, Hassan G, Rousseau C, Pottie K, Canadian Collaboration for Immigrant and Refugee Health (CCIRH) (2011). Common mental health problems in immigrants and refugees: general approach in primary care. CMAJ.

[20] Sundquist J (2001). Migration, equality and access to health care services. J Epidemiol Community Health.

[21] Karnoe A, Kayser L (2015). How is eHealth literacy measured and what do the measurements tell us? a systematic review. Knowledge Management E-Learning.

[22] Diviani N, Dima AL, Schulz PJ (2017). A psychometric analysis of the Italian version of the eHealth Literacy Scale using item response and classical test theory methods. J Med Internet Res.

[23] Tomás C, Queirós P (2014). Analysis of the psychometric properties of the portuguese version of an eHealth literacy assessment tool. Revista de Enfermagem Referência.

[24] Chang A, Schulz PJ (2018). The measurements and an elaborated understanding of Chinese eHealth literacy (C-eHEALS) in chronic patients in China. Int J Environ Res Public Health.

[25] Mitsutake S, Shibata A, Ishii K, Okazaki K, Oka K (2011). [Developing Japanese version of the eHealth Literacy Scale (eHEALS)]. Nihon Koshu Eisei Zasshi.

[26] Paige SR, Krieger JL, Stellefson M, Alber JM (2017). eHealth literacy in chronic disease patients: An item response theory analysis of the eHealth literacy scale (eHEALS). Patient Educ Couns.

[27] Paramio PG, Almagro BJ, Hernando GÁ, Aguaded GJI (2015). [Validation of the eHealth Literacy Scale (eHEALS) in Spanish University Students]. Rev Esp Salud Publica.

[28] van der Vaart Rosalie, van Deursen Alexander Jam, Drossaert CH, Taal E, van Dijk Jan Amg, van de Laar Mart Afj (2011). Does the eHealth Literacy Scale (eHEALS) measure what it intends to measure? validation of a Dutch version of the eHEALS in two adult populations. J Med Internet Res.

[29] Brørs G, Wentzel-Larsen T, Dalen H, Hansen TB, Norman CD, Wahl A, Norekvål TM, CONCARD Investigators (2020). Psychometric properties of the Norwegian version of the Electronic Health Literacy Scale (eHEALS) among patients after percutaneous coronary intervention: cross-sectional validation study. J Med Internet Res.

[30] Richtering SS, Morris R, Soh S, Barker A, Bampi F, Neubeck L, Coorey G, Mulley J, Chalmers J, Usherwood T, Peiris D, Chow CK, Redfern J (2017). Examination of an eHealth literacy scale and a health literacy scale in a population with moderate to high cardiovascular risk: Rasch analyses. PLoS One.

[31] Koo M, Norman CD, Hsiao-Mei C (2012). Psychometric evaluation of a Chinese version of the eHealth literacy scale (eHEALS) in school age children. Int Electron J Health Educ.

[32] Wangdahl JM, Dahlberg K, Jaensson M, Nilsson U (2019). Psychometric validation of Swedish and Arabic versions of two health literacy questionnaires, eHEALS and HLS-EU-Q16, for use in a Swedish context: a study protocol. BMJ Open.

[33] Mokkink LB, Prinsen CA, Patrick DL, Alonso J, Bouter LM, de Vet HCW, Terwee CB (2019). COSMIN study design checklist for patient-reported outcome measurement instruments. COSMIN.

[34] Terwee CB, Bot SDM, de Boer Michael R, van der Windt Daniëlle A W M, Knol DL, Dekker J, Bouter LM, de Vet Henrica C W (2007). Quality criteria were proposed for measurement properties of health status questionnaires. J Clin Epidemiol.

[35] Guillemin F, Bombardier C, Beaton D (1993). Cross-cultural adaptation of health-related quality of life measures: literature review and proposed guidelines. J Clin Epidemiol.

[36] Valerio MA, Rodriguez N, Winkler P, Lopez J, Dennison M, Liang Y, Turner BJ (2016). Comparing two sampling methods to engage hard-to-reach communities in research priority setting. BMC Med Res Methodol.

[37] Mokkink LB, Terwee CB, Patrick DL, Alonso J, Stratford PW, Knol DL, Bouter LM, de Vet Henrica C W (2010). The COSMIN study reached international consensus on taxonomy, terminology, and definitions of measurement properties for health-related patient-reported outcomes. J Clin Epidemiol.

[38] VanVoorhis C, Morgan B (2007). Understanding power and rules of thumb for determining sample sizes. Tutor Quant Methods Psychol.

[39] Bujang M, Baharum N (2017). A simplified guide to determination of sample size requirements for estimating the value of intraclass correlation coefficient: a review. Arch Orofac Sci.

[40] Sørensen Kristine, Van den Broucke Stephan, Pelikan JM, Fullam J, Doyle G, Slonska Z, Kondilis B, Stoffels V, Osborne RH, Brand H, HLS-EU Consortium (2013). Measuring health literacy in populations: illuminating the design and development process of the European Health Literacy Survey Questionnaire (HLS-EU-Q). BMC Public Health.

[41] Nielsen SS, Krasnik A (2010). Poorer self-perceived health among migrants and ethnic minorities versus the majority population in Europe: a systematic review. Int J Public Health.

[42] Benyamini Yael (2011). Why does self-rated health predict mortality? an update on current knowledge and a research agenda for psychologists. Psychol Health.

[43] de Vet HC, Terwee CB, Mokkink LB, Knol DK (2014). Measurement in Medicine: A Practical Guide.

[44] Berkman ND, Sheridan SL, Donahue KE, Halpern DJ, Crotty K (2011). Low health literacy and health outcomes: an updated systematic review. Ann Intern Med.

[45] Palumbo R, Annarumma C, Adinolfi P, Musella M, Piscopo G (2016). The Italian Health Literacy Project: insights from the assessment of health literacy skills in Italy. Health Policy.

[46] Sørensen Kristine, Pelikan JM, Röthlin Florian, Ganahl K, Slonska Z, Doyle G, Fullam J, Kondilis B, Agrafiotis D, Uiters E, Falcon M, Mensing M, Tchamov K, van den Broucke Stephan, Brand H, HLS-EU Consortium (2015). Health literacy in Europe: comparative results of the European health literacy survey (HLS-EU). Eur J Public Health.

[47] Paasche-Orlow MK, Parker RM, Gazmararian JA, Nielsen-Bohlman LT, Rudd RR (2005). The prevalence of limited health literacy. J Gen Intern Med.

[48] Hälleberg Nyman Maria, Nilsson U, Dahlberg K, Jaensson M (2018). Association between functional health literacy and postoperative recovery, health care contacts and health related quality of life among patients undergoing day surgery. JAMA Surg.

[49] Easton P, Entwistle VA, Williams B (2010). Health in the 'hidden population' of people with low literacy. a systematic review of the literature. BMC Public Health.

[50] Levin-Zamir D, Baron-Epel OB, Cohen V, Elhayany A (2016). The association of health literacy with health behavior, socioeconomic indicators, and self-assessed health from a national adult survey in Israel. J Health Commun.

[51] Almaleh R, Helmy Y, Farhat E, Hasan H, Abdelhafez A (2017). Assessment of health literacy among outpatient clinics attendees at Ain Shams University Hospitals, Egypt: a cross-sectional study. Public Health.

[52] Ng E, Omariba DWR (2013). Immigration, generational status and health literacy in Canada. Health Educ J.

[53] de Winter JCF, Dodou D (2015). Common factor analysis versus principal component analysis: a comparison of loadings by means of simulations. Commun Stat Simul Comput.

[54] Schober P, Boer C, Schwarte LA (2018). Correlation coefficients: appropriate use and interpretation. Anesth Analg.

[55] Cronbach LJ (1951). Coefficient alpha and the internal structure of tests. Psychometrika.

[56] Landis JR, Koch GG (1977). The measurement of observer agreement for categorical data. Biometrics.

[57] Parkvall M (2018). Arabiska Sveriges näst största modersmål [Arabic Sweden's second largest mother tongue]. Svenska Dagbladet.

[58] Kalfoss M (2019). Translation and adaption of questionnaires: a nursing challenge. SAGE Open Nurs.

[59] Chung S, Nahm E (2015). Testing reliability and validity of the eHealth Literacy Scale (eHEALS) for older adults recruited online. Comput Inform Nurs.

[60] Chung S, Park BK, Nahm E (2018). The Korean eHealth Literacy Scale (K-eHEALS): reliability and validity testing in younger adults recruited online. J Med Internet Res.

[61] Lin C, Broström A, Griffiths MD, Pakpour AH (2019). Psychometric evaluation of the Persian eHealth Literacy Scale (eHEALS) among elder Iranians with heart failure. Eval Health Prof.

[62] Juvalta S, Kerry MJ, Jaks R, Baumann I, Dratva J (2020). Electronic health literacy in Swiss-German parents: cross-sectional study of eHealth literacy scale unidimensionality. J Med Internet Res.

[63] Ma Z, Wu M (2019). The psychometric properties of the Chinese eHealth literacy scale (C-eHEALS) in a Chinese rural population: cross-sectional validation study. J Med Internet Res.

[64] Neter E, Brainin E, Baron-Epel O (2015). The dimensionality of health literacy and eHealth literacy. Eur Health Psychologist.

