# Supplementary material for: Arabic Version of the Electronic Health Literacy Scale in Arabic-Speaking Individuals in Sweden: Prospective Psychometric Evaluation Study
Source: J Med Internet Res. 2021 Mar 22;23(3):e24466. doi: 10.2196/24466 (PMC8074986; doi:10.2196/24466)
Supplement: Multimedia Appendix 1 [file jmir_v23i3e24466_app1.docx]

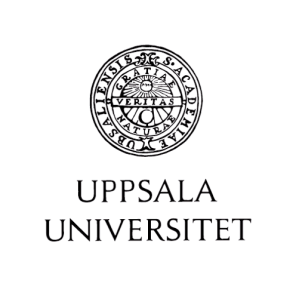

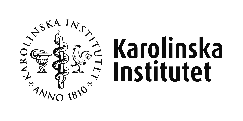


**أسئلة** **حول** **المعلومات** **الصحية** **على** **شبكة** **الإنترنت**

| لكل عبارة أدناه، أي أجابة تتوافق أحسن مع رأيك أو تجربتك في الوقت الحاضر  أشر على الخيار الذي يطابق إجابتك الأحسن في كل سطر. | | | | | |
| --- | --- | --- | --- | --- | --- |
|  | لا أوافق أبداً | لا أوافق ً | لست متأكد | أوافق | أوافق تماما |
| **1**. أنا اعرف **أي من المعلومات الصحية** متواجدة على الإنترنت |  |  |  |  |  |
| 2. أنا أعرف **أين** أَجِد المعلومات الصحية المفيدة **على الإنترنت** |  |  |  |  |  |
| 3. أنا أعرف **كيفية** العثور (الوصول) على المعلومات الصحية المفيدة على الانترنت |  |  |  |  |  |
| 4. أنا **أعرف كيف أستخدم الإنترنت** للإجابة على أسئلتي التي تتعلق بالصحة |  |  |  |  |  |
| 5. أنا أعرف **كيف أستخدم المعلومات الصحية** التي اجدها على الإنترنت لمساعدتي |  |  |  |  |  |
| 6. لدي المهارات اللازمة **لتقييم** ما إذا كانت المعلومات الصحية التي أجدها على الإنترنت يمكن الوثوق بها |  |  |  |  |  |
| 7. أنا أستطيع التمييز بين المعلومات الصحية ذات **الجودة العالية** وبين المعلومات الصحية ذات **الجودة المنخفضة** التي أجدها على الإنترنت |  |  |  |  |  |
| 8. **أنا اثق بقدرتي** في إستخدام المعلومات التي أجدها على الإنترنت لإتخاذ قرارات حول الصحة |  |  |  |  |  |

**شكرا** **جزيلا** **لإجابتك** **على** **الأسئلة**!
